# Cytomorphological Effects of Mitomycin C on Urothelial Cells: Eosinophils May Be Clue to The Drug-Induced Changes

**Published:** 2014-10-04

**Authors:** Gulcin Guler Simsek, Erdem Vargol, Hulya Simsek

**Affiliations:** Kecioren Training and Research Hospital, Department of Pathology Ankara, Turkey

**Keywords:** Mitomycine C, Urothelial Cell, Eosinophil

## Abstract

Cytomorphological changes of mitomycin C on urothelial cells may be misinterpreted as a neoplastic process. A 60-year old male patient who was given an eight-week course of intravesical mitomycin C due to non-invasive low grade transitional cell carcinoma. During his follow-up care, the findings of a urine cytology exam were as follows: nuclear enlargement of cells, wrinkled nuclear membranes, little hyperchromasia, pleomorphism, abnormal nuclear morphology and disordered orientation of the urothelium. Furthermore, there were eosinophils nearby the atypical cells. This report aimed at reminding the cytomorphologic changes of mitomycin C may be misinterpreted as carcinoma, so the presence of eosinophils is required to predict the drug-induced changes.

## Introduction

Alkylating agents such as mitomycin C can result in atypical changes in nuclei of urothelial cells that mimic carcinoma. These agents used as topical therapy tend to have an abrasive effect on papillary tumors, resulting in structures that can be misdiagnosed as flat lesions (1). They can cause interpretation problems in cytologic findings and biopsy materials. This case is reported to draw the attention to that of carcinoma mimicker process.

## Case report

**Fig 1:**
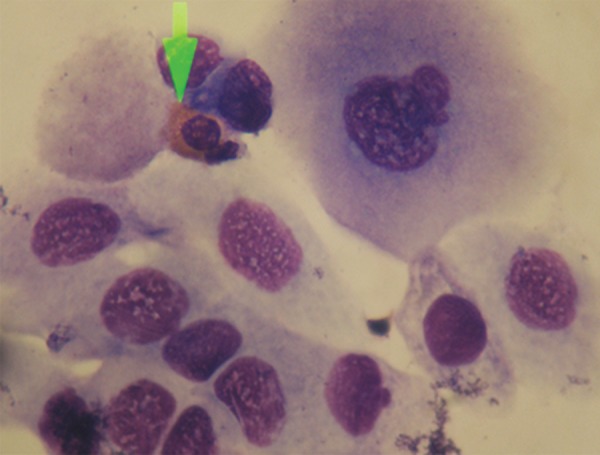
Eosinophil found near the atypical hyperchromatic urothelial cells with irregular nuclear membrane and voluminous cytoplasm (MGG, ×100).

A 60-year old male patient presented with the complaint of hematuria. Thinking as bladder tumor by cystoscopic examination, transurethral resection (TUR) was performed. Through histopathological findings, he was diagnosed with noninvasive low grade transitional cell carcinoma (Ta TCC). He was given an eight-week course of intravesical mitomycin C. During his follow-up care, urine cytology was beneficial due to the following factors: atypical cells with nuclear enlargement, wrinkled nuclear membranes, and little hyperchromasia nuclei, distinct or multiple nucleoli. Furthermore, there were eosinophils nearby the atypical cells in the material (Fig 1). Suspicious urinary cytology was reported. The cystoscoy detected a hyperemic area seen at the contact of the anterior wall and the dome. Punch biopsy results from that area identified the following factors: low degree pleomorphism , abnormal nuclear morphology and disordered orientation of the urothelium. In some area, surface epithelium was detached, but in some other areas, there was superficial maturation of the epithelium. Based on histological and cytological findings, these cytomorphologic changes were due to adjuvant therapy, mitomycin C, applied for the patient.

This report aimed at reminding the cytomorphologic changes of mitomycin C may be misinterpreted as carcinoma (in situ), so review of the literature and presence of eosinophiluria are required for a proper identification of the drug-induced changes.

## Discussion

Superficial bladder cancers are responsible for 70 to 80% of all newly diagnosed bladder cancers. Superficial tumors include carcinoma in situ (CIS), tumors confined to the mucosal epithelium (Ta), and superficial tumors invading the lamina propria (T1), without involvement of superficial muscle layers. The primary treatment for eradication of stage Ta and T1 bladder cancers is TUR of the tumor. Many patients with superficial bladder tumors undergoing endoscopic surgery alone have shown recurrence or tumor progression at some point in their follow-up care, while some superficial tumors (15 to 25%) are at high risk for progression to muscle invasion. The requirement for adjuvant treatment becomes a major attention in these early tumors (2, 3).

Cytopathological examination of urinary specimens is considered as a leading method for detecting and for monitoring patients with bladder lesions. The limitations of cystoscopy and of biopsy for monitoring bladder cancer patients increase the need for urinary cytology, which is crucial for those having carcinoma in situ or receiving topical therapy. The best type of urinary specimen for this method is freshly voided, randomly collected urine. Catheterised urines and bladder washing specimens yield more and better preserved cells (4). Bladder wash cytology has been usually applied to detect the recurrences since majority of the patients with positive cytology and endoscopy, respectively, develop a recurrent tumor (5).

Mitomycin C and other alkylating agents produce characteristic of cell lines with nonspecific changes in urothelial cells that may mimic those of carcinoma (4). These drugs affect mostly superficial umbrella cells, causing enlargement of the nucleus and cell. The nuclei are round to oval in shape, moderately enlarged and multiple. The nuclear membranes are usually smooth, but may be wrinkled due to degeneration (crenation). Nuclei usually have smudgy-appearing chromatin, while multiple small nucleoli are common. The cytoplasm is degenerated, vacuolated, and frayed (6). Significant cytologic atypia should be carefully investigated.

Eosinophils in urinary cytology are associated with bladder cancer in some cases, while these cells may also be induced by drugs. Among the most common causes of eosinophiluria together with other leukocytes, urinary tract infection, bladder injury and acute interstitial nephritis have been detected mainly.

In our case, presence of eosinophils together with atypical cells may be clue to drug-altered urothelial cells. The effects of mitomycin C are very similar to those previously reported for thio-tepa. Murphy et al. have indicated that these chemicals produce drugspecific light microscopic alterations. Mitomycin C and thio-tepa behave as toxic substances, causing increased exfoliation, degeneration, and necrosis of urothelial cells (7, 8). Although all of these drugs have toxicity, they usually are well tolerated (3).

Mitomycin C can result in nuclear changes, especially in superficial urothelial cells that mimic carcinoma. So, interpretation of carcinoma in urinary samples should rely upon changes in non-superficial urothelial cells that are not significantly affected by therapy. Presence of eosinophils may also lead to proper diagnosis of the drug-induced changes.
